# Plasma Proteome Profiling of Coronary Artery Disease Patients: Downregulation of Transthyretin—An Important Event

**DOI:** 10.1155/2020/3429541

**Published:** 2020-11-10

**Authors:** Monu, Rupsi Kharb, Ankita Sharma, Monu Kumar Chaddar, Rakesh Yadav, Prachi Agnihotri, Anand Kar, Sagarika Biswas

**Affiliations:** ^1^Council of Industrial Research (CSIR)-Institute of Genomics and Integrative Biology, Mall Road, Delhi University Campus, 110007, Delhi, India; ^2^Delhi Institute of Pharmaceutical Sciences and Research (DIPSAR), University of Delhi, Pushpvihar, New Delhi 110017, India; ^3^All India Institute of Medical Sciences, Ansari Nagar, New Delhi 110029, India; ^4^School of Life Sciences, Takshashila Campus, Devi Ahilya University, 452017, Indore, India

## Abstract

Coronary artery disease (CAD) is a prevalent chronic inflammatory cardiac disorder. An early diagnosis is likely to help in the prevention and proper management of this disease. As the study of proteomics provides the potential markers for detection of a disease, in the present investigation, attempt has been made to identify disease-associated differential proteins involved in CAD pathogenesis. For this study, a total of 200 selected CAD patients were considered, who were recruited for percutaneous coronary intervention (PCI) treatment. The proteomic analysis was performed using two-dimensional gel electrophoresis (2-DE) and MALDI-TOF MS/MS. Samples were also subjected to Western blot analysis, enzyme-linked immunosorbent assay (ELISA), peripheral blood mononuclear cells isolation immunofluorescence (IF) analysis, analytical screening by fluorescence-activated cell sorting (FACS), and in silico analysis. The representative data were shown as mean ± SD of at least three experiments. A total of 19 proteins were identified. Among them, the most abundant five proteins (serotransferrin, talin-1, alpha-2HS glycoprotein, transthyretin (TTR), fibrinogen-*α* chain) were found to have altered level in CAD. Serotransferrin, talin-1, alpha-2HS glycoprotein, and transthyretin (TTR) were found to have lower level, whereas fibrinogen-*α* chain was found to have higher level in CAD plasma compared to healthy, confirmed by Western blot analysis. TTR, an important acute phase transport protein, was validated low level in 200 CAD patients who confirmed to undergo PCI treatment. Further, in silico and in vitro studies of TTR indicated a downexpression of CAD in plasma as compared to the plasma of healthy individuals. Lower level of plasma TTR was determined to be an important risk marker in the atherosclerotic-approved CAD patients. We suggest that the TTR lower level predicts disease severity and hence may serve as an important marker tool for CAD screening. However, further large-scale studies are required to determine the clinical significance of TTR.

## 1. Introduction

Coronary artery disease (CAD) is causing high rate of mortality and morbidity in the modern world. It is characterized by arterial blockage through deposition of cholesterol and/or other material on inner wall of arteries which lead to develop a plaque called atherosclerosis [1]. It can remain stable for some years or can induce the formation of an occlusive coronary thrombus at the site of atherosclerotic plaque and rupture and lead to the development of cardiovascular events, such as unstable angina or myocardial infarction, i.e., acute coronary syndromes (ACS) [2]. It was observed that about 17.7 million (31%) global deaths occurred due to cardiovascular diseases (CVD). Among these cases, deaths because of CAD and strokes were 7.4 million and 6.7 million, respectively, as estimated by the WHO (WHO 2017) [3]. Since CAD is a complex disorder where both genetic, and lifestyle especially, the dietary habits can influence the heart health, understanding disease pathophysiology, and finding new diagnostic biomarker for easy and early detection of CAD that is imperative.

Molecular biomarkers have become increasingly important clinical tools for CAD screening and diagnosis. Presently, the C-reactive protein (CRP) has been widely used as an established nonspecific prognostic inflammatory biomarker for the prediction and diagnosis of CAD [4, 5]. Other prevailing biomarkers for CAD diagnosis include interleukin-6 (IL-6) [6] and tumor necrosis factor-alpha (TNF-*α*) [7]. Moreover, the multimarker approach such as troponin-I and B-type natriuretic peptide (BNP) in combination also showed improved risk stratification. Although few genetic markers such as single nucleotide polymorphisms (SNPs) have also been identified but are not clinically used to diagnose CAD, most of them could not be replicated in different populations. Also, these identified candidate proteins are very difficult to detect in the plasma, which is the most readily available clinical sample [8]. Therefore, we have used plasma for the identification of predictive biomarkers for CAD assessment. It has been shown that the changes in plasma protein levels could promote plaque (cholesterol-laden) deposition in arteries causing their hardening and promoting pathogenesis [9, 10]. Earlier also, studying these plasma proteins could provide diverse and integrated information about the disease mechanisms that contribute to CAD pathophysiology [11]. In fact, some plasma biomarkers have been identified by high-throughput mass spectrometry (MS) analysis [12] and have been reported as diagnostic or therapeutic biomarkers for various diseases such as cancer, neurological diseases, and autoimmune diseases [13]. Although advances in proteomic analysis led to the identification of few markers for CAD diagnosis [4], till date, hardly anyone is known to be highly specific to diagnose CAD at an early stage [14]. Hence, there is an urgent need for a specific marker protein in the prospect of CAD diagnosis.

Previous studies have shown that protein phosphorylation manages regulation of cell proliferation, differentiation, apoptosis, protein subcellular localization, and complex formation through different signalling pathways and diverse functions, depending upon the occupation of various phosphorylation sites [15]. About one-third of mammalian proteins are phosphorylated and many pathological conditions are characterized by these altered phosphorylation levels during disease states. Previously, a variety of cardiovascular pathologies have demonstrated that changes in the phosphorylation states of key cardiac regulatory proteins may underlie cardiac dysfunction in disease states [16]. In general, phosphorylation of specific residues on target substrates triggers small conformational changes in the protein structure which can alter the biological function. It has been well established that protein phosphorylation is intimately involved in the regulation of myocardial contractility, metabolism, and other processes like membrane transport and permeability and ionic fluxes, regulated by this versatile posttranslational mechanism [17, 18]. Therefore, addressing the abundance of protein phosphorylation could be a useful resource to understand the molecular mechanism to target CAD pathogenesis.

In this context, we aimed to identify phosphoproteins along with differential abundance of proteins in CAD. Therefore, the identification of phosphoproteins from CAD plasma was employed using the phosphoprotein enrichment technique. It was also thought that the identification of differential proteins using a phosphoproteomics approach at the preliminary stage of CAD may help to understand their disease association particularly in the cohort of patient samples [19]. Thus, we have identified the differential level of phosphoproteins that might have pathogenesis relevance and serve as predictive protein markers for CAD.

Among all identified protein, transthyretin (TTR) has been validated to elucidate its differential level consistency in CAD because TTR is well known as an acute-phase reactant protein, whose wild-type transthyretin amyloidosis variant expression has been observed to be reduced during the inflammatory response of familial amyloidotic polyneuropathy (FAP) and biopsy-proven amyloidosis [20]. It appears to have a diverse pathophysiological impact on the cardiovascular health. TTR, often known as prealbumin, a plasma protein with a tetramer molecule of 56 kDa, normally synthesized in the liver and choroid plexus of the brain and is secreted into the bloodstream and cerebrospinal fluid [21]. However, further comprehensive evaluation of TTR required in larger cohort of CAD may hold its clinical importance and will potentially pave a path to reveal the regulatory mechanism of CAD.

## 2. Materials and Methods

### 2.1. Sample Collection

For this study, 200 CAD patients from the All India Institute of Medical Sciences (AIIMS), New Delhi, India, were enrolled who were having multivessel stenosis, as affirmed by the coronary CT angiography. The patients with age of 18 years and/or above were included for study, whereas the patients having a history of heart failure, myocardial infarction (MI), renal failure, atrial fibrillation, coronary artery bypass grafted (CABG), and pregnant women were excluded from this study.

From each patient, the blood sample was taken prior to the percutaneous coronary intervention (PCI). The pathological status of each patient was confirmed at the department of cardiology of the same institute. The clinical information along with definite medical history of each patient, their lifestyle characteristics including age, symptoms, and the prior history of cardiovascular related disorder such as acute coronary syndrome (ACS), stroke and hypertension (H/T), and diabetes mellitus (DM) were also recorded (Table 1). Similarly, healthy control (*n* = 50) individuals having no history of prior cardiac ailments with the same age group were considered. Written and signed consents were obtained from all volunteers before enrolment. The study was carried out in accordance with the principles of the Helsinki Declarations and approved by the medical ethics committee of Department of Cardiology, AIIMS, New Delhi, India (reg. No. IEC/NP-252/2013), and CSIR-Institute of Genomics and Integrative Biology, Mall Road, Delhi University Campus, Delhi, India.

### 2.2. Sample Processing

The blood samples from CAD patients and healthy individuals were drawn using venepuncture in an ethylene diamine tetra acetic acid- (EDTA-) coated vacutainer tubes (BD, Franklin Lakes, NJ, USA) and transported immediately in a cold chamber to the research premises. Blood samples were centrifuged at 1300 × g for 15 min at 4°C, and plasma was separated and processed immediately for running 2-DE and other experiments. The remaining plasma samples were aliquoted in various volumes in eppendorf and stored at -80°C for further analysis [11].

### 2.3. Phosphoprotein Enrichment

The phosphoproteins from CAD and healthy control plasma were enriched using a phosphoprotein enrichment kit (Thermo Scientific, Pierce, USA) in triplicate using manufacturer's protocol as described earlier [22]. The columns were loaded with resin and equilibrated with exchange buffer. Plasma samples (CAD *n* = 5 and healthy *n* = 5) were pooled separately and used for phosphoprotein enrichment in order to minimize intraindividual heterogeneity. A total of 0.5-1.0 mg protein was taken from each pooled sample. The plasma samples were then exchanged with 0.5 M triethylammonium bicarbonate (TEAB, pH 8.5) buffer using 3 kDa cut-off filter (Millipore, USA). The fractions of phosphoproteins were eluted using elution buffer and quantitated by the Bradford assay.

### 2.4. Two-Dimensional Gel Electrophoresis (2-DE)

The enriched phosphoproteins pooled samples of CAD and healthy control were used to run 2-DE in triplicates as described previously with slight modifications [22]. The main advantages of 2-DE are its ability to analyze the complete protein at high resolution with good separation, apart from robustness and reproducibility. A total of 70 *μ*g phosphoprotein-enriched samples of CAD and healthy plasma were dissolved separately in rehydration buffer [7 M urea, 2 M thiourea, 2% 3-[(3-Cholamidopropyl) dimethylammonio]-1-persulfonate (CHAPS), 0.2% (v/v) ampholytes, and bromophenol blue] were applied to 7 cm immobilized pH gradient strips (IPG) ranging 4-7 pH (Bio-Rad, Munich, Germany) and rehydrated overnight. The first dimension by isoelectric focusing (IEF) was carried out at 200 V for 1 h and 500 V for 1 h followed by final focusing at 8000 V for 5 h. Before the second dimension, IPG strips were equilibrated in buffer (50 mM Tris-HCl, pH 8.8, 6 M Urea, 4% (w/v) SDS, 20% (w/v) glycerol and 10 mg/ml DTT) and reequilibrated in the same buffer containing 40 mg/ml iodoacetamide (IAA) replacing DTT, each for 20 min. The equilibrated strips were run in 12% sodium dodecyl sulfate-polyacrylamide gel electrophoresis (SDS-PAGE) using the mini protean-II electrophoresis vertical system (Bio-Rad, Munich, Germany) for 2 h at 100 V constant voltage. The gels were phosphostained using Pro-Q Diamond and silver staining of gel for total proteins (Merck Chemicals, Germany). The gel spots thus obtained were scanned using the chemiDoc™ MP imaging system (Bio- Red, USA) [23].

### 2.5. Phosphoprotein Staining

For phosphoproteome analysis, the gels were stained using Pro-Q Diamond phosphoprotein stain (Molecular Probes, Invitrogen, UK) according to the manufacturer's instruction with little modification. Briefly, 2-DE gels were first fixed twice in 100 ml of 50% methanol, 10% acetic acid for 30 min each, and washed with Milli Q. The gels were incubated with Pro-Q Diamond for phosphoprotein staining for 2 h at room temperature (RT) and destained with 20% acetonitrile (ACN) and 50 mM sodium acetate for 30 min followed by washing thrice with Milli Q. After washing, gels were scanned using FLA-5100 (Fuji photo film, Co. Ltd: Europa GmbH, Dusseldorf, Germany) with excitation and emission maxima 532 nm and 580 nm, respectively. Later, silver staining of the same gels was carried out and scanned using the chemiDocTM MP imaging system (Bio- Red, USA) as described earlier [22].

### 2.6. 2-DE Image Analysis

The gels were analyzed using PDQuest image lab software (Bio-Rad). The normalized protein amount for each spot was calculated as the ratio of the spot volume to the toral spot volume. A threshold level for differential protein level was defined as at least 1.5-fold increase or decrease in spot intensity between the groups.

### 2.7. In Gel Trypsin Digestion

Protein spots were excised and trypsin-digested as described previously with some modifications [24]. The gel spots were destained with 30 mM potassium ferricyanide (Sigma Aldrich, USA) and 100 mM sodium thiosulphate (Sigma Aldrich, USA) and dehydrated with acetonitrile (ACN)/water until they become white and sticky. The gel pieces were equilibrated using 100 mM ammonium bicarbonate and ACN (Sigma Aldrich, USA), for 5 min and vacuum dried. The gel pieces were digested with sequencing grade trypsin 10 *μ*l (0.1 *μ*g/*μ*l trypsin, Promega, USA) overnight at 37°C. Following digestion, tryptic peptides were extracted twice with 50% ACN/0.1% trifluoroacetic acid (TFA, Sigma Aldrich, USA) with moderate sonication, dried in a speed-vac to expel TFA/ACN, and stored at -20°C till further use.

### 2.8. MALDI-TOF MS/MS Analysis

The molecular masses of polypeptides were determined using matrix-assisted laser desorption ionization mass spectrometer (MALDI-TOF MS/MS) (Applied Biosystems, Life Technologies, USA). The extracted peptides were desalted and purified using ZipTip C18 (Millipore). Peptide (1 *μ*l) concentrate was mixed with 1 *μ*l sinapinic acid onto the MALDI target plate and allowed to air dry. MS/MS spectra were procured over the mass scope of 10,000-20,000 Da in reflector positive mode with 5600 laser shots force (25 shots/subspectrum for 500 aggregate shots/spectrum). The inside alignment was performed utilizing a standard calibration mix 5 (Applied Biosystems). For the MS/MS precursor selection, the least S/N (signal/noise) filter was set at 25 with a prohibition list for *α*-cyano-4-hydroxycinnamic acid (CHCA) lattice peaks. Protein identification was considered statistically significant probability-based score (*p* ≤ 0.05) using the mascot database search [25].

### 2.9. Western Blot Analysis

The plasma protein (20 *μ*g) from CAD and healthy controls was separated on 12% SDS-PAGE as described previously [25] and electrotransferred to the nitrocellulose (NC) membrane (Millipore, USA) using Trans-Blot Semi-dry transfer unit (Bio-Rad, USA). The membranes were blocked using 5% of bovine serum albumin (BSA) (Sigma-Aldrich, USA) for 1 h at RT. Thereafter, NC membranes were incubated overnight at 4°C with their respective primary antibodies: serotransferrin, talin-1, *α*-2HS glycoprotein, TTR ,and fibrinogen-*α* chain with 1 : 4000 dilution and with horseradish peroxidase- (HRP-) conjugated anti-mouse secondary antibody (Jackson, USA), at RT for 1 h with 1 : 8000 dilutions on the gentle shaking condition. Each membrane was then developed with enhanced chemiluminescence (ECL) (Thermo Scientific, Pierce, USA), a highly sensitive substrate detector using the Chemi-Doc_TM_ MP Imaging system (Bio-Rad, USA). After each incubation, membranes were washed thrice with wash buffer (0.05% tween-20 in a phosphate buffer saline).

### 2.10. Enzyme-Linked Immunosorbent Assay Analysis

The enzyme-linked immunosorbent assay (ELISA) was carried out using anti-TTR antibody (ProSci, USA). Plasma samples were diluted (1 *μ*l/200 *μ*l) using coating buffer comprising sodium carbonate (Na2CO3, 0.01 M) and sodium bicarbonate (NaHCO3, 0.035 M), pH 9.6. The diluted samples were dispensed into 96 well microtiter plates (Thermo Scientific, Nunc, USA) and incubated overnight at 4°C. The plates were washed thrice using washing buffer containing 0.1% tween-20 in a phosphate buffer saline (PBS), blocked by 1% of bovine serum albumin (BSA, Sigma-Aldrich, USA), and incubated for 1 h at RT. After blocking, wells were washed and incubated with diluted (1 : 2000) primary anti-TTR antibody (ProSci, USA) for 2 h at RT. Followed by washing, wells were washed and incubated with diluted (1 : 1000) HRP-conjugated secondary anti-mouse antibody (Jackson, USA) at RT for 1 h. The reactions were developed using orthophenylene diamine (1 mg/ml) and stopped by adding 3 N H2SO4. The absorbances were observed at 495 nm via ELISA reader (Spectra Max Plus 384, molecular devices) as demonstrated previously [25].

### 2.11. Peripheral Blood Mononuclear Cell Isolation

The peripheral blood mononuclear cells (PBMCs) from blood of CAD and healthy control were isolated using the density gradient reagent. The fresh blood was overlaid on to a density gradient medium histopaque (Sigma-Aldrich, USA) at RT and centrifuged at 1500 × g for 30 min at 25°C by setting acceleration and deceleration rate at 9 and 3, respectively. PBMCs were aspirated carefully and diluted in Dulbecco's Modified Eagle's Medium (DMEM) containing 10% fetal bovine serum (FBS) in 1 : 1 ratio. After centrifugation at 1500 × g for 10 min at 25°C, the supernatant was decanted, and pellet was washed with PBS pH 7.4, centrifuged again at 1500 × g for 10 min and resuspended into 50 *μ*l of 1X PBS for further use [26].

### 2.12. Analytical Screening by Fluorescence-Activated Cell Sorting (FACS)

To detect the expression of TTR, the PBMCs from CAD and healthy control were first washed with 1X PBS and incubated with 200 *μ*l of cytoperm (BD, Bioscience, US) fixative for 25 min at 4°C. After centrifugation at 3000 × g for 5 min at RT, nonspecific binding was blocked with 1.5% BSA (Sigma-Aldrich, USA) for 30 min at 4°C in the dark. The cells were stained with diluted (1 : 1000) anti-TTR antibody (Santa Cruz Biotech., inc.) and incubated for 2 h at 4°C. Followed by washing, cells were allowed to react with secondary antibody goat anti-Mouse IgG (H + L), Alexa Fluor® 647 conjugated (Make-Invitrogen), and TTR-positive stained PBMCs were observed in the FACS machine (FACS Calibur BD Bioscience). After each step, the cells were centrifuged and washed thrice using 200 *μ*l 1X PBS [27].

### 2.13. Immunofluorescence (IF) Analysis

To examine the localization of TTR, CAD and healthy control PBMCs were smeared on poly-L-lysine-coated glass slides and air dried. The cells were fixed using 2% paraformaldehyde (PFA) for 15 min at RT. Further, 0.2% triton-x-100 (Sigma, Life Science) was applied to permeabilized cells for 15 min, and nonspecific binding was blocked with 2% BSA (Sigma-Aldrich, USA) for 30 min at RT. Followed by washing, cells were incubated overnight at 4°C with diluted (1 : 100) primary anti-human TTR monoclonal antibody (Santa Cruz Biotech., inc.) and then with diluted (1 : 200) secondary antibody anti-mouse IgG (H + L) Alexa Fluor® 546 conjugated (Make-Invitrogen) for 1 hr at RT. The nuclei of cells were stained using the 4′,6-diamidino-2-phenylindole (DAPI) reagent onto the slides and air dried in the dark at RT. The stained immune cells were examined under confocal fluorescence microscopy (Leica CRT 6500 fluorescence microscopy, USA). Samples without anti-TTR were used as a control for background staining [27].

### 2.14. In Silico Analysis of TTR

Site localization studies of TTR were carried out using the SubLoc server (http://www.bioinfo.tsinghua.edu.cn/SubLoc/) [28]. This server is a localization annotation database having >6000 sequences of proteins. In this server, predictions are made with the help of the support vector machine (SVM) and protein subcellular localization prediction tool (PSORT II) program. The PSORT II server (http://psort.hgc.jp/form2.html) was also employed in order to determine the TTR location. The updated version of this server is known as WoLF PSORT. This server changes the query protein sequence in numerical localization features. Converted sequence of the protein was predicted using the k-nearest neighbor classifier [29], and the MultiLoc server (http://abi.inf.uni-tuebingen.de/Services/MultiLoc/) was utilized to predict the location of TTR [30]. In the MultiLoc server, predictions are made on the basis of signal peptide, sequence specific motif, and composition of amino acid from the already established motif database. SVM used these sequence features to predict the site of localization. Expected accuracy of the server in the crossvalidation test was found to be ∼75%. Also, the “STRING” pathway online tool was used to find out the interacting partners of TTR.

### 2.15. Statistical Analysis

For statistical data analysis, representative data are shown as mean ± SD, and the unpaired *t*-test was used considering *p* ≤ 0.05 as statistically significant of at least three experiments. Using receiver operating characteristics (ROC) area under curve (AUC) was calculated to distinguish healthy control and CAD individual for true positive and false positive results.

## 3. Results

### 3.1. 2-DE Analysis and Protein Identification by MALDI-TOF MS/MS

**Figure 1:**
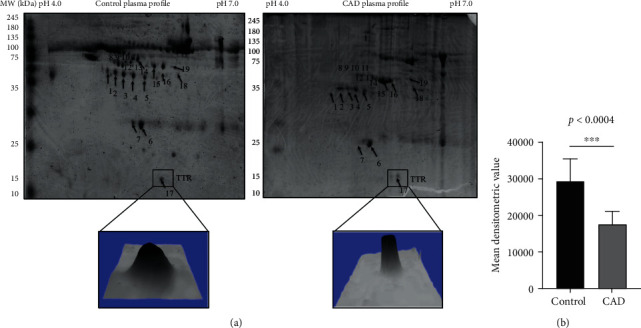
Two-dimensional gel electrophoresis of plasma proteins. (a) Representative 2-DE gel image of plasma proteins and 3D view distinctly indicates the spot intensity of TTR CAD plasma, compared to the healthy control. (b) TTR densitometry analysis indicated low level in CAD with significant difference 1.7-fold *p* < 0.0004 in comparison to control. The protein spot normalized to the total spot intensity in the gel. The threshold level for the differential protein level was considered as at least 1.5-fold increase or decrease in spot intensity that was significant at the *p* < 0.05 level using Student's *t*-test. TTR: transthyretin; CAD: coronary artery disease.

In the preliminary phase, the clinical demography of CAD patients (*n* = 200) has been presented in Table 1. From the results of the 2-DE gel study (Figure 1), a total of 26 protein spots were detected followed by image analysis using PDQuest image lab software (Bio-Rad) and analyzed densitometrically. The protein spots were marked and normalized to the total spot intensity in the gel. A threshold of at least 1.5-fold increase or decrease in spot intensity between the group was considered significant at the *p* < 0.05 level. The significant spots were excised for identification. Among 26 protein spots, 19 spots were successfully identified by MALDI-TOF MS/MS and the mascot search database (Table 2). It was observed that several spots of the same accession number were identified for one protein, probably isoforms resulting from an amino acid substitution and/or posttranslation modification. Our results thus identified 10 distinct proteins including keratin type-I cytoskeletal 10 (spot, 1), hemopexin (spot, 2 and 3), haptoglobin (spot, 4 and 5), fibrinogen gamma chain (spot, 6), serotransferrin (spot, 7), alpha1antitrypsin (spot, 8-11), fibrinogen alpha chain (spot, 12 and 13), keratin type- I cytoskeletal 10 (spot, 14,15), apo-lipoprotein A-I (spot, 16), transthyretin (spot, 17), talin-1 (spot, 18), and alpha-2HS glycoprotein (spot, 19) from CAD plasma (Table 2).

Compared to earlier proteomic studies, the identified proteins are known to be associated with cardiovascular diseases and are described in discussion. Among all the identified proteins, densitometric analysis of the TTR protein spot detected was 1.7-fold lower level (*p* < 0.0004) (Figure 1(b)) in CAD compared to healthy plasma after normalization described above. Interestingly, a good peptide matches were obtained across with the percentage sequence coverage by mass spectrometry analysis (Suppl. Fig S1).

### 3.2. Western Blot Analysis of Significant Altered Protein

**Figure 2:**
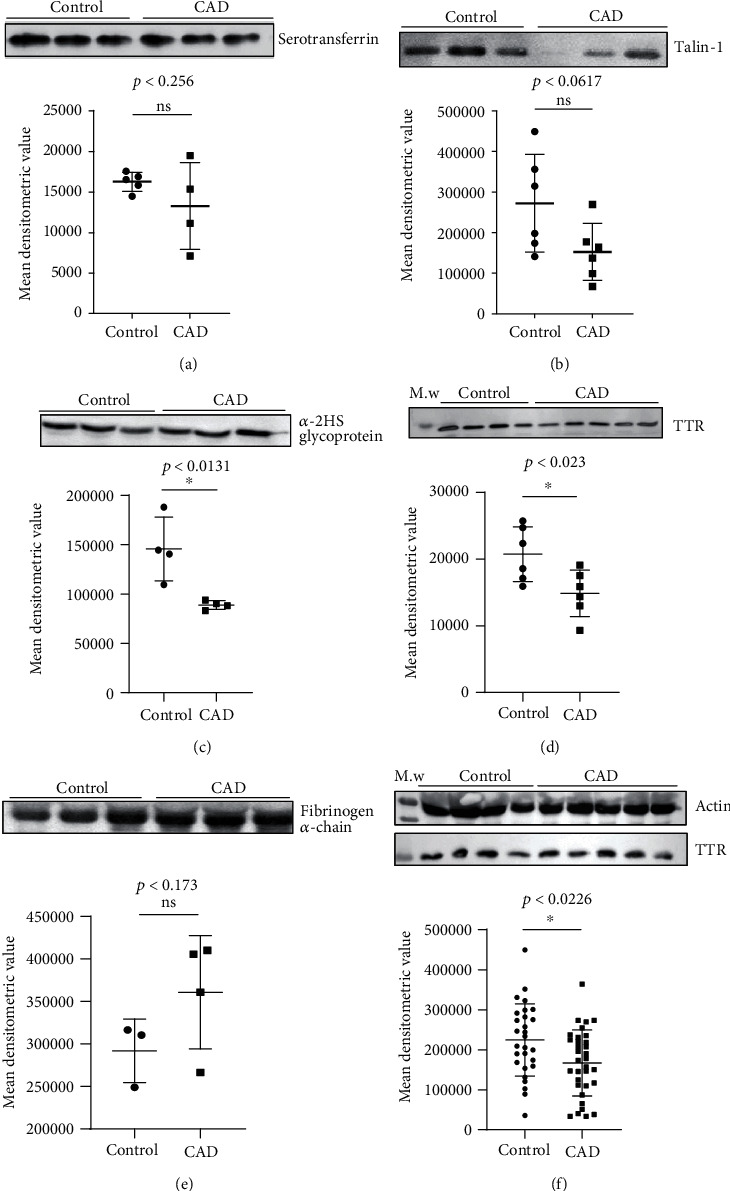
Validation of differential protein level analysis in CAD plasma. Western blot analysis representing altered level of distinctly identified proteins from CAD plasma. Serotransferrin protein (a), Talin-1 protein (b), Alpha-2HS glycoprotein (c), and TTR (d) showed low level with a statistical difference of 1.1-fold, *p* < 0.256; 1.45-fold, *p* < 0.0617; 1.31-fold, *p* < 0.0131; and 1.5-fold, *p* < 0.023, respectively, in CAD (*n* = 5) compared healthy control (*n* = 5 in each). However, the fibrinogen- *α* chain protein (e) showed high level in CAD (*n* = 5) plasma with a statistical difference of 1.2-fold, *p* < 0.173 compared to healthy control. Similarly, low level of TTR (f) was observed in CAD (*n* = 50) plasma compared to healthy control (*n* = 50). Densitometric analysis of band intensities indicates a statistical difference of 1.4-fold, *p* < 0.0001, after normalization with actin loading control in CAD plasma compared to healthy control. Data represent mean ± SD, and statistical significance is determined by Student's *t*-test, *p* < 0.05.

The most abundant five out of ten distinct proteins were considered based upon their spot intensity analysis. For initial confirmation and validation, CAD individual patients (*n* = 5) and healthy control (*n* = 5) plasma samples were screened of the same age and sex to nullify age-dependent and/or age-dependent comorbidities. The differential protein level was validated in few (*n* = 5) individual samples by Western blotting, and densitometric analysis was carried out by using Image Lab 5.1 software (Bio-Rad). Results revealed lower level of four proteins with fold change including serotransferrin 1.1-fold, *p* < 0.256 (Figure 2(a)), talin-1 1.45-fold, *p* < 0.0617 (Figure 2(b)), *α*-2HS glycoprotein 1.31-fold, *p* < 0.0131 (Figure 2(c)), transthyretin 1.5-fold, *p* < 0.023 (Figure 2(d)), and higher level of fibrinogen alpha chain with a fold change of 1.2-fold, *p* < 0.173 (Figure 2(e)). *p* value thus indicates that except TTR and *α*-2HS glycoprotein proteins, other 3 proteins were statistically nonsignificant.

Among these two statistically significant low-level differential proteins, TTR was further confirmed and validated to determine its significant low-level consistency among randomly picked *n* = 50 CAD patients' plasma in comparison to *n* = 50 healthy control plasma. The densitometric analysis followed by Western blot revealed a significant lower level of TTR in more CAD samples that showed a statistical difference of 1.4-fold, *p* < 0.022 (Figure 2(f)), after normalizing with actin as a loading control. The second significantly differential candidate protein, alpha-2HS glycoprotein, has been reported to be lower level in the plasma of CAD and has been found to be associated with a higher mortality risk in CAD patients [31].

### 3.3. Validation of Differentially Expressed TTR

**Figure 3:**
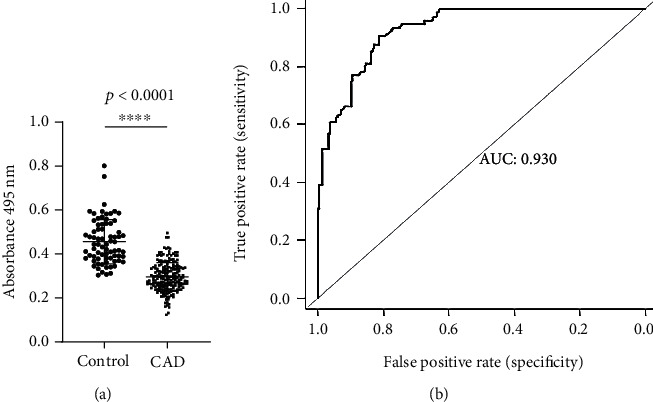
Validation of TTR in CAD Plasma: (a) The enzyme-linked immunosorbent assay of TTR from plasma of CAD patients (*n* = 200) and control (*n* = 50) indicated significant low level with a 1.61-fold *p* < 0.0001. (b) The ROC plot indicates the individual protein abundance of each group with area under curve AUC: 0.930. The statistical significance was determined by Student's *t*-test, *p* < 0.05.

**Figure 4:**
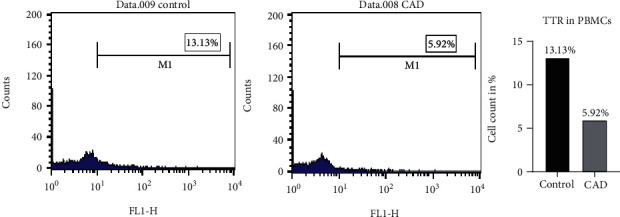
FACS histogram of the TTR protein expression in the whole blood cell population: observed percentage of the TTR protein expression in CAD (5.92%) compared to healthy control (13.13%) indicates the 2-fold downexpression in CAD patients by calculating the number of cells out of the parent population (total cells) that showed positive staining after gating the unstained cell population.

To further confirm the result of TTR in CAD plasma, ELISA and FACS analysis was carried out. Validation of TTR by ELISA in CAD (*n* = 200, 170 male and 30 female) plasma showed lower level with a significant difference of 1.61-fold, (*p* < 0.0001) (Figure 3(a)) compared to healthy control. The receiver operating characteristic curve (ROC) graph was obtained to confirm the true positive against the false positive, and the area under curve (AUC) value was observed to be 0.93 (Figure 3(b)). FACS analysis was carried out by comparing healthy and CAD stained PBMCs. The percentage value for TTR positive cells was calculated by the number of cells out of the parent population (total cells) that showed positive staining after gating the unstained cell population in the FACS dot plot on FL4 window, and the gated cell population was measured on FL1 using the histogram peaks. The level of TTR confirms 2-fold lower levels in CAD PBMCs with a percentage protein expression of 5.92% as against 13.13% of healthy controls (Figure 4).

### 3.4. TTR Interaction and Localization Analysis

The analysis of interacting partner provided a detailed insight into the functional significance of TTR in association with other proteins using STRING [32]. The predicted protein-protein interaction (PPI) of TTR partner proteins includes apolipoprotein A-I that have importance in reverse transport of cholesterol from tissues to the liver and acting as a cofactor for the lecithin cholesterol acyltransferase (LCAT), apolipoprotein-IV contributes in chylomicrons and very low-density lipoprotein (VLDL) secretion and catabolism, retinol binding protein-4 (RBP4) delivers retinol from the liver and stores to the peripheral tissues, heparan sulfate proteoglycan-2 (HSPG2) interacts with lipid metabolism by binding with lipoprotein lipase (LpL), and apolipoprotein-B (Apo-B) has been reported as a major constituent of atherogenic lipoproteins and plays a significant role in association with high-density lipoprotein (HDL) and low-density lipoprotein (LDL). This PPI analysis suggests that TTR has an associative role with other disease-associated proteins which are profoundly well known (Suppl. Fig S2), and understanding this may highlight that TTR contributes to shared functions in disease state.

The site of TTR localization was therefore identified using the SubLoc server and revealed that cytoplasm is the localization site of TTR with reliability index-1 and expected accuracy 56%. The MultiLoc server also indicated that peroxisome and cytoplasm as the probable sites of localization (Suppl. Table 1). The results were verified by the -II server that showed TTR is having cytoplasmic, nuclear, and cytoskeleton localizations with accuracy of 60.9%, 13%, and 8.7%, respectively (Suppl. Table 2). In silico analysis was further confirmed by the localization site using immunofluorescence analysis resulting into the conclusion that apart from plasma, TTR shows varied localization after analyzing total positive cell intensities with respect to the healthy cells.

The overall positive stained cell intensity showed low expression of TTR within CAD patient PBMC compared to healthy control's total PBMC intensity (Suppl. Fig 4). The changes in the intracellular TTR expression might cause its functional inference in CAD. Though localization study requires further investigation to comprehend the TTR role, it can be established further to understand the cellular mechanism responsible for the downexpression of TTR.

## 4. Discussion

Early diagnosis of CAD is essential to prevent damage occurring in the arteries during the plaque formation [33]. Report shows that abnormal regulation of posttranscription, posttranslation, and phosphorylation of proteins is responsible for deterioration of a disease condition, and understanding those abnormal protein phosphorylation could be used as a pathological hallmark of diseases [34]. Thus, in our preliminary studies, we have identified 10 distinct altered phosphoproteins from CAD plasma, among which Apo-A1, hemopexin, haptoglobin, and Alpha-antitrypsin have been reported to be associated with acute coronary syndrome [35]. The other 5 proteins including serotransferrin, talin-1, *α*-2HS glycoprotein, fibrinogen alpha chain, and transthyretin not being studied well with respect to CAD considered for further validation in CAD plasma. As the molecular events in CAD are diverse and remain to be clearly understood, identification of these altered proteins in angiography-approved CAD patient's plasma may add value in understanding their role in disease.

Serotransferrin, an iron binding transporter protein, involved in tumor development, promotes endothelial cell migration and invasion [36]. Its lower level in rheumatic valvular disease (RVD) patients' plasma advances pathogenicity [37], similar to our studies indicating that serotransferrin may have association with CAD.

Talin-1, an important structural protein, mediates integrin activation [38] and maintains immune homeostasis [39]. Lower level of Talin-1 in CVD is associated with proliferation of vascular smooth muscle cells [40, 41] and may lead to the CAD progression as per present results.

Alpha-2HS, phosphorylated glycoprotein/fetuin-A, is known to participate in the antifibrotic activity, and its lower levels found to be associated with impaired coronary flow in ST-segment elevation myocardial infarction patients [42] apart from playing multiple roles in diabetes mellitus, CVDs, and rheumatoid arthritis [25, 31, 43]. Likewise, its lower level in our studies may limit the cardiac function, hence disease progression.

Fibrinogen, an acute-phase proinflammatory mediator, elevated in CVD, involves in blood clotting [44] and is also elevated in CAD similar to our studies, associated with atherosclerosis [45].

TTR, carrier protein, binds to retinol (vitamin A) and thyroxin (*T*_4_) to facilitate the biological function [21]. Role of TTR remains elusive in CAD pathophysiology. To our knowledge, till now, there is no/negligible proteomic study regarding identification of the phosphorylated protein in combination of analyzing the differential proteome of CAD and validation of TTR in 200 samples collected prior to PCI treatment. However, lower level of serum prealbumin causing adverse cardiac event in existed ACS patients was observed earlier [46]. Also, different levels of TTR in other disease like familial amyloid [47], senile systemic amyloidosis and schizophrenia [48], and its altered level in human carotid atherosclerotic tissues were also reported [49]. But the underlying mechanism of altered level in disease remains to be revealed. Our study revealed low level of TTR in CAD plasma by 2-DE, Western, ELISA, FACS, and by in silico analysis. ROC analysis determines the variability in the TTR level among CAD individuals, and the flow cytometry analysis confirmed 2-fold lower level of TTR in PBMCs isolated from CAD plasma (Figure 4). Thus, the altered pattern of TTR at cellular levels may specify it as a disease indicator, suggesting that its alter behavior might have an associative role with circulating monocytes and lymphocyte cells.

It is known that proteins can interact directly or indirectly via sharing a metabolic pathway or by regulating each other transcriptionally for functional signification [32]. Therefore, to elucidate the predictive functional importance of TTR, protein-protein interaction (PPI) was performed using STRING analysis indicating that TTR is sharing interaction with the apolipoprotein family that are profoundly associated with CAD (Suppl. Fig S2) and plays a key role in cholesterol transport mechanism [50, 51]. Additionally, we have also observed that, apart from plasma, the altered level of TTR in cytoplasm together with different numbers of TTR-positive stained cells (Suppl. Fig S4) may indicate an important event in CAD pathogenesis.

Although localization study requires further investigation to comprehend the TTR role, the immunofluorescence (IF) result (supple. Fig 4) confirmed the predictive presence of TTR in different cells, may provide a clue towards the involvement of TTR in particular cell subtypes of PBMCs, and might have an association with CAD. Localization abundance of TTR in CAD may also be possible due to its receptor-mediated uptake and intracellular interaction with apolipoprotein A-I (Apo-A1) via high-density lipoprotein (HDL) or low-density lipoprotein (LDL) [52]. TTR possesses secretary property, and its sequence analysis indicated the presence of signal peptide that is confined to a cleavage site and may introduce amyloid formation (Suppl. Fig S3). It is also reported to bind and destabilized the Apo A-I structure, promoting cholesterol efflux from cells causing aggregation and/or amyloid depositions and inducing cytotoxicity [50]. It might have occurred due to consequent dissociation and variation in the TTR structure, causing extracellular proteinaceous deposition preferentially in cardiac tissues and coronary arteries [53, 54]. Evidently, altogether, it may cause alteration in the TTR level, and analyzing this may partake functional implication in CAD disease.

Despite several studies, the molecular pathway between TTR and its differential mechanism in angiography-approved CAD is still obscure. As we have observed low plasma TTR in CAD patients who has undergone PCI treatment, it may have its own clinical significance, although the validation with larger sample size is necessary to define the precise role of TTR in CAD pathophysiology to enlighten a novel insight into the disease mechanisms.

## 5. Conclusion

In the current study, consistent and significant low level of TTR in angiography-approved 200 CAD plasma may predict disease severity. It is therefore suggested that low level of TTR requires further investigation in larger sample size to determine the clinical significance that may consider to serve as a tool for CAD screening and therapeutic target.

## Figures and Tables

**Table 1:** Demography clinical assessment of CAD patients and healthy individuals. CAD: coronary artery disease; D/M: diabetes mellitus; H/T: hypertension; CABG: coronary artery bypass grafting; ACS: acute coronary syndrome; PCI: percutaneous coronary intervention.

S. no.	Characteristics	CAD patients (*n* = 200)	Healthy individuals (*n* = 50)
1	Meanage ± SD	56.5 ± 10.40	50.1 ± 6.05
2	Sex (male, female)	170M+30F	36M+14F
3	D/M (%)	65 (32.5%)	0 (0)
4	H/T (%)	103 (51.5%)	0 (0)
5	Past history of CABG (%)	0 (0)	0 (0)
6	Past history of PCI (%)	8 (4%)	0 (0)
7	Past history of stroke (%)	3 (1.5%)	0 (0)
8	Past history of ACS (%)	104 (52%)	0 (0)
9	Medicine	Antiplatelet, nitrates, *β*-blocker, statins, etc.	Nil

**Table 2:** List of identified proteins from MALDI-TOF MS/MS analyses, which are differentially expressed in plasma of CAD patients with respect to healthy control. For statistical evaluation, *p* < 0.05 was used as a significance threshold during the Mascot search database and uniProt database (http://www.uniprot.org/) that were searched for the protein function. Annotation: MALDI-TOF MS/MS: matrix-assisted laser desorption/ionization mass spectrometry; M.W.: molecular weight; Da: Dalton.

Spot no.	Proteins identified	Accession no.	M.W. (Da)	Peptide matched	Score	Biological functions
1	Keratin type I cytoskeletal 10	P13645	59020	4	88	Structural constituent of epidermis, cornification, keratinization, and keratinocyte differentiation.
2	Hemopexin	P02790	52385	3	75	Cellular iron ion homeostasis, Hemoglobin metabolic process, and metal ion binding.
3	Hemopexin	P02790	52385	3	136	Cellular iron ion homeostasis, along with the heme metabolic process and metal ion binding.
4	Haptoglobin	P00738	45861	3	75	Antioxidant activity and hemoglobin-binding protein involved in the acute-phase response and defense response. Additionally, the vital role in negative regulation of the oxidoreductase activity has been noted.
5	Haptoglobin	P00738	45861	4	91	Antioxidant activity and hemoglobin-binding protein involved in the acute-phase response and defense response. Additionally, the vital role in negative regulation of the oxidoreductase activity has been noted.
6	Fibrinogen alpha chain	P02671	94971	3	92	Blood coagulation, cell-matrix adhesion, cellular protein complex assembly, response to calcium ion.
7	Serotransferrin	P02787	79294	2	57	Cellular iron ion homeostasis and import ferrous iron into the cell. Additionally, transferrin regulates protein stability and transport transferrin.
8	Alpha-1-anti trypsin	P01009	46878	3	47	It is a well-known inhibitor of serine proteases and has a moderate affinity for plasmin and thrombin. Additionally, it inhibits trypsin, chymotrypsin, and plasminogen activator.
9	Alpha-1-anti trypsin	P01009	46878	4	107	It is a well-known inhibitor of serine proteases and has a moderate affinity for plasmin and thrombin. Additionally, it inhibits trypsin, chymotrypsin, and plasminogen activator.
10	Alpha-1-anti trypsin	P01009	46878	3	90	It is a well-known inhibitor of serine proteases and has a moderate affinity for plasmin and thrombin. Additionally, it inhibits trypsin, chymotrypsin, and plasminogen activator.
11	Alpha-1-anti trypsin	P01009	46878	3	61	It is a well-known inhibitor of serine proteases and has a moderate affinity for plasmin and thrombin. Additionally, it inhibits trypsin, chymotrypsin, and plasminogen activator.
12	Fibrinogen-alpha chain	P02671	94971	2	70	Fibrin has a major function in hemostasis as one of the primary components of blood clots and wound repair. Additionally, it also guides cell migration during reepithelialization.
13	Fibrinogen-gamma chain	P02679	52106	2	59	Blood coagulation, cell-matrix adhesion, cellular protein complex assembly, response to calcium ion.
14	Keratin type I cytoskeletal 10	P13645	59020	8	255	It is a structural constituent of epidermis and cornification that plays role in keratinization and keratinocyte differentiation.
15	Keratin type I cytoskeletal 10	P13645	59020	4	88	It is a structural constituent of epidermis and cornification that plays role in keratinization and keratinocyte differentiation.
16	Apolipoprotein A-I	P02647	30759	16	98	The major contribution of apo family proteins has been reported in cholesterol biosynthetic and metabolic process and also involves in high-density lipoprotein particle modelling.
17	Transthyretin	P02766	15991	5	80	It is involved in the cellular and retinoid metabolic process with dual binding ability. It has also organized extracellular matrix, identical protein binding, and thyroid hormone binding activities.
18	Talin-1	Q9Y490	240120	26	45	Talin-1 plays varied function such as actin filament binding, cadherin binding, integrin binding, phosphatidylinositol binding, and phosphatidylserine binding.
19	Alpha-2HS-glycoprotein	P02765	64214	12	36	Alpha-2HS-glycoprotein has the potential to involved in the kinase inhibitor activity, cellular protein metabolic process, regulation of inflammatory response, platelet degranulation, and neutrophil degranulation.

## Data Availability

Data availability and data supporting the findings of this study are available from the corresponding author [Dr. Sagarika Biswas] on request. The patient's samples were obtained from the Department of Cardiology, AIIMS, New Delhi, India (reg. No. IEC/NP-252/2013), after the ethical clearance for the current study.

## Supplementary Materials

Supplementary Materials The details regarding have been represented such as graph of the MALDI mascot score distribution and immunofluorescence analysis of TTR expression and localization. The in silico analysis (*Supplementary materials*).

## References

[1] Mosterd A., Hoes A. W. (2007). Clinical epidemiology of heart failure. *Heart*.

[2] Assmann G., Schulte H. (1992). Relation of high-density lipoprotein cholesterol and triglycerides to incidence of atherosclerotic coronary artery disease (the PROCAM experience). *The American Journal of Cardiology*.

[3] Benjamin E. J., Blaha M. J., Chiuve S. E. (2017). American Heart Association Statistics Committee and Stroke Statistics Subcommittee. Heart Disease and Stroke Statistics-2017 Update: A Report From the American Heart Association. *Circulation*.

[4] Wang J., Tan G. J., Han L. N., Bai Y. Y., He M., Liu H. B. (2017). Novel biomarkers for cardiovascular risk prediction. *Journal of Geriatric Cardiology*.

[5] Auer J., Berent R., Lassnig E., Eber B. (2002). C-reactive protein and coronary artery Disease. *Japanese Heart Journal*.

[6] Su D., Li Z., Li X. (2013). Association between serum interleukin-6 concentration and mortality in patients with coronary artery disease. *Mediators of Inflammation*.

[7] Hua X. P., Zhang X. D., Kwong J. S. W., Zeng X. T., Zhang Z. J., Wei W. L. (2015). Tumor necrosis factor- alpha G-238A polymorphism and coronary artery disease risk: a meta-analysis of 4, 222 patients and 4, 832 controls. *Therapeutics and Clinical Risk Management*.

[8] LiI T. Y., Tse M. Y., Ventura N. M., Hetu M.-F., Johri A. M., Pang S. C. (2018). Abstract 162: single nucleotide polymorphism of the B-type natriuretic peptide helps predict the presence of significant coronary artery disease. *Circulation Research*.

[9] Napoli C., D'Armiento F. P., Mancini F. P. (1997). Fatty streak formation occurs in human fetal aortas and is greatly enhanced by maternal hypercholesterolemia. Intimal accumulation of low density lipoprotein and its oxidation precede monocyte recruitment into early atherosclerotic lesions. *Journal of Clinical Investigation*.

[10] Liu W., Zhao Y., Wu J. (2018). Gene expression profile analysis of the progression of carotid atherosclerotic plaques. *Molecular Medicine Reports*.

[11] Basak T., Tanwar V. S., Bhardwaj G. (2016). Plasma proteomic analysis of stable coronary artery disease indicates impairment of reverse cholesterol pathway. *Scientific Reports*.

[12] Beck H. C., Overgaard M., Melholt Rasmussen L. (2015). Plasma proteomics to identify biomarkers – application to cardiovascular diseases. *Translational Proteomics*.

[13] Frangogiannis N. G. (2012). Biomarkers: hopes and challenges in the path from discovery to clinical practice. *Translational Research*.

[14] Röthlisberger S., Pedroza-Diaz J. (2017). Urine protein biomarkers for detection of cardiovascular disease and their use for the clinic. *Expert Review of Proteomics*.

[15] Liu Y., Chance M. R. (2014). Integrating phosphoproteomics in systems biology. *Computational and structural biotechnology*.

[16] Li S. M., Liu W. T., Yang F., Yi Q. J., Zhang S., Jia H. L. (2019). Phosphorylated proteomics analysis of human coronary artery endothelial cells stimulated by Kawasaki disease patients serum. *BMC Cardiovascular Disorders*.

[17] Ardito F., Giuliani M., Perrone D., Troiano G., Muzio L. L. (2017). The crucial role of protein phosphorylation in cell signaling and its use as targeted therapy (review). *International Journal of Molecular Medicine*.

[18] Kuzmanov U., Guo H., Buchsbaum D. (2016). Global phosphoproteomic profiling reveals perturbed signaling in a mouse model of dilated cardiomyopathy. *Proceedings of the National Academy of Sciences of the United States of America*.

[19] Subirana I., Fitó M., Diaz O. (2018). Prediction of coronary disease incidence by biomarkers of inflammation, oxidation, and metabolism. *Scientific Reports*.

[20] Gruys E., Toussaint M. J. M., Niewold T. A., Koopmans S. J. (2005). Acute phase reaction and acute phase proteins. *Journal of Zhejiang University Science*.

[21] Van Jaarsveld P. P., Edelhoch H., Goodman D. S., Robbins J. (1973). The interaction of human plasma retinol-binding protein and prealbumin. *The Journal of Biological Chemistry*.

[22] Zahid S., Oellerich M., Asif A. R., Ahmed N. (2012). Phosphoproteome profiling of substantia nigra and cortex regions of Alzheimer’s disease patients. *Journal of Neurochemistry*.

[23] Saroha A., Biswas S., Chatterjee B. P., Das H. R. (2011). Altered glycosylation and expression of plasma alpha-1-acid glycoprotein and haptoglobin in rheumatoid arthritis. *Journal of Chromatography B*.

[24] Panda S., Kar A., Biswas S. (2017). Preventive effect of Agnucastoside C against isoproterenol- induced myocardial injury. *Scientific Reports*.

[25] Biswas S., Sharma S., Saroha A. (2013). Identification of novel autoantigen in the synovial fluid of rheumatoid arthritis patients using an immunoproteomics approach. *PLoS One*.

[26] Panda S. K., Ravindran B. (2013). Isolation of human PBMCs. *Bio-Protocol*.

[27] Pattnaik B., Bodas M., Bhatraju N. K. (2016). IL-4 promotes asymmetric dimethylarginine accumulation, oxo-nitrative stress, and hypoxic response-induced mitochondrial loss in airway epithelial cells. *The Journal of Allergy and Clinical Immunology*.

[28] Chen H., Huang N., Sun Z. (2006). SubLoc: a server/client suite for protein subcellular location based on SOAP. *Bioinformatics*.

[29] Horton P., Park K. J., Obayashi T. (2007). WoLF PSORT: protein localization predictor. *Nucleic Acids Research*.

[30] Hoglund A., Donnes P., Blum T., Adolph H. W., Kohlbacher O. (2006). MultiLoc: prediction of protein subcellular localization using N-terminal targeting sequences, sequence motifs and amino acid composition. *Bioinformatics*.

[31] Chen X., Zhang Y., Chen Q., Li Q., Li Y., Ling W. (2017). Lower plasma fetuin-a levels are associated with a higher mortality risk in patients with coronary artery disease. *Arteriosclerosis, Thrombosis, and Vascular Biology*.

[32] Franceschini A., Szklarczyk D., Frankild S. (2012). STRING v9.1: protein-protein interaction networks, with increased coverage and integration. *Nucleic Acids Research*.

[33] Lygirou V., Latosinska A., Makridakis M. (2018). Plasma proteomic analysis reveals altered protein abundances in cardiovascular disease. *Journal of Translational Medicine*.

[34] Thomas S. N., Cripps D., Yang A. J. (2009). Proteomic analysis of protein phosphorylation and ubiquitination in Alzheimer's disease. *Methods in Molecular Biology*.

[35] Tan Y., Liu T. R., Hu S. W. (2014). Acute coronary syndrome remodels the protein cargo and functions of high-density lipoprotein subfractions. *PLoS One*.

[36] Carlevaro M. F., Albini A., Ribatti D. (1997). Transferrin promotes endothelial cell migration and invasion: implication in cartilage neovascularization. *The Journal of Cell Biology*.

[37] Gao G., Xuan C., Yang Q., Liu X. C., Liu Z. G., He G. W. (2013). Identification of altered plasma proteins by proteomic study in valvular heart diseases and the potential clinical significance. *PLoS One*.

[38] Park E. J., Yuki Y., Kiyono H., Shimaoka M. (2015). Structural basis of blocking integrin activation and deactivation for anti-inflammation. *Journal of Biomedical Science*.

[39] Klann J. E., Remedios K. A., Kim S. H. (2017). Talin plays a critical role in the maintenance of the regulatory T cell pool. *Journal of Immunology*.

[40] Wei X., Sun Y., Wu Y. (2017). Downregulation of Talin-1 expression associates with increased proliferation and migration of vascular smooth muscle cells in aortic dissection. *BMC Cardiovascular Disorders*.

[41] Manso A. M., Okada H., Sakamoto F. M. (2017). Loss of mouse cardiomyocyte talin-1 and talin-2 leads to *β*-1 integrin reduction, costameric instability, and dilated cardiomyopathy. *Proceedings of the National Academy of Sciences of the United States of America*.

[42] Basar N., Sen N., Kanat S. (2011). Lower fetuin-a predicts angiographic impaired reperfusion and mortality in ST-elevation myocardial infarction. *Journal of Investigative Medicine*.

[43] Sun Z. L., Xie Q. Y., Guo G. L., Ma K., Huang Y. Y. (2014). Serum fetuin-a levels in patients with cardiovascular disease: a meta-analysis. *BioMed Research International*.

[44] Kazmi R. S., Lwaleed B. A. (2012). Plasminogen and fibrinogen plasma levels in coronary artery disease. *Revista Brasileira de Hematologia e Hemoterapia*.

[45] Davalos D., Akassoglou K. (2012). Fibrinogen as a key regulator of inflammation in disease. *Seminars in Immunopathology*.

[46] Wang W., Wang C.-S., Ren D., Li T., Yao H.-C., Ma S.-J. (2018). Low serum prealbumin levels on admission can independently predict in-hospital adverse cardiac events in patients with acute coronary syndrome. *Medicine*.

[47] Molina G. O., Judge D., Campbell W., Chahal H., Mugmon M. (2014). Transthyretin cardiac amyloidosis: an under-diagnosed cause of heart failure. *Journal of Community Hospital Internal Medicine Perspectives*.

[48] Saraiva M. J. M., Sherman W., Marboe C. (1990). Cardiac amyloidosis: report of a patient heterozygous for the transthyretin isoleucine 122 variant. *Scandinavian Journal of Immunology*.

[49] Liang W., Ward L. J., Karlsson H. (2016). Distinctive proteomic profiles among different regions of human carotid plaques in men and women. *Scientific Reports*.

[50] Liz M. A., Gomes C. M., Saraiva M. J., Sousa M. M. (2007). ApoA-I cleaved by transthyretin has reduced ability to promote cholesterol efflux and increased amyloidogenicity. *Journal of Lipid Research*.

[51] Sousa M. M., Berglund L., Saraiva M. J. (2000). Transthyretin in high density lipoproteins: association with apolipoprotein A-I. *Journal of Lipid Research*.

[52] Sousa M. M., Saraiva M. J. (2001). Internalization of Transthyretin. *Journal of Biological Chemistry*.

[53] Marques L. R., Diniz T. A., Antunes B. M. (2018). Reverse cholesterol transport: molecular mechanisms and the non-medical approach to enhance HDL cholesterol. *Frontiers in Physiology*.

[54] Teng M. H., Yin J. Y., Vidal R. (2001). Amyloid and nonfibrillar deposits in mice transgenic for Wild-Type human transthyretin: a possible model for senile systemic amyloidosis. *Laboratory Investigation*.

