# Supplementary material for: Plasma Proteome Profiling of Coronary Artery Disease Patients: Downregulation of Transthyretin—An Important Event
Source: Mediators Inflamm. 2020 Nov 10;2020:3429541. doi: 10.1155/2020/3429541 (PMC7707994; doi:10.1155/2020/3429541)
Supplement: Supplementary Materials — The details regarding have been represented such as graph of the MALDI mascot score distribution and immunofluorescence analysis of TTR expression and localization. The in silico analysis (Supplementary materials). [file 3429541.f1.docx]

# Plasma proteome profiling of coronary artery disease patients

# : Down-regulation of transthyretin, an important event

**Monu^1^, Rupsi Kharb^1, 2^, Ankita Sharma^1^, Monu Kumar Chaddar^1^, Rakesh Yadav^3^, Prachi Agnihotri^1^, Anand Kar^4^, Sagarika Biswas^1^***

^1^ Department of Genomics & Molecular Medicine, CSIR-Institute of Genomics and Integrative Biology, New Delhi, India,110007

^2^ Department of pharmaceutics, Delhi Institute of Pharmaceutical Sciences and Research (DIPSAR), University of Delhi, Pushpvihar, New Delhi, India

^3^ Department of Cardiology, All India Institute of Medical Sciences, New Delhi, India

^4^School of Life Sciences, Takshashila Campus, Devi Ahilya University, Indore, India,452017

***Corresponding author**

Dr. Sagarika Biswas

Department of Genomics & Molecular Medicine

Institute of Genomics and Integrative Biology (CSIR),

Mall Road, Delhi-110 007, India

Tel: +91 11 27667602

& Fax #: +91-11-27667471,#9818004740

E-mail: [sagarika.biswas@igib.res.in](mailto:sagarika.biswas@igib.res.in)

**Supplementary Materials**

**Supplementary Results and Tables legends**

**Supplementary Figure 1**


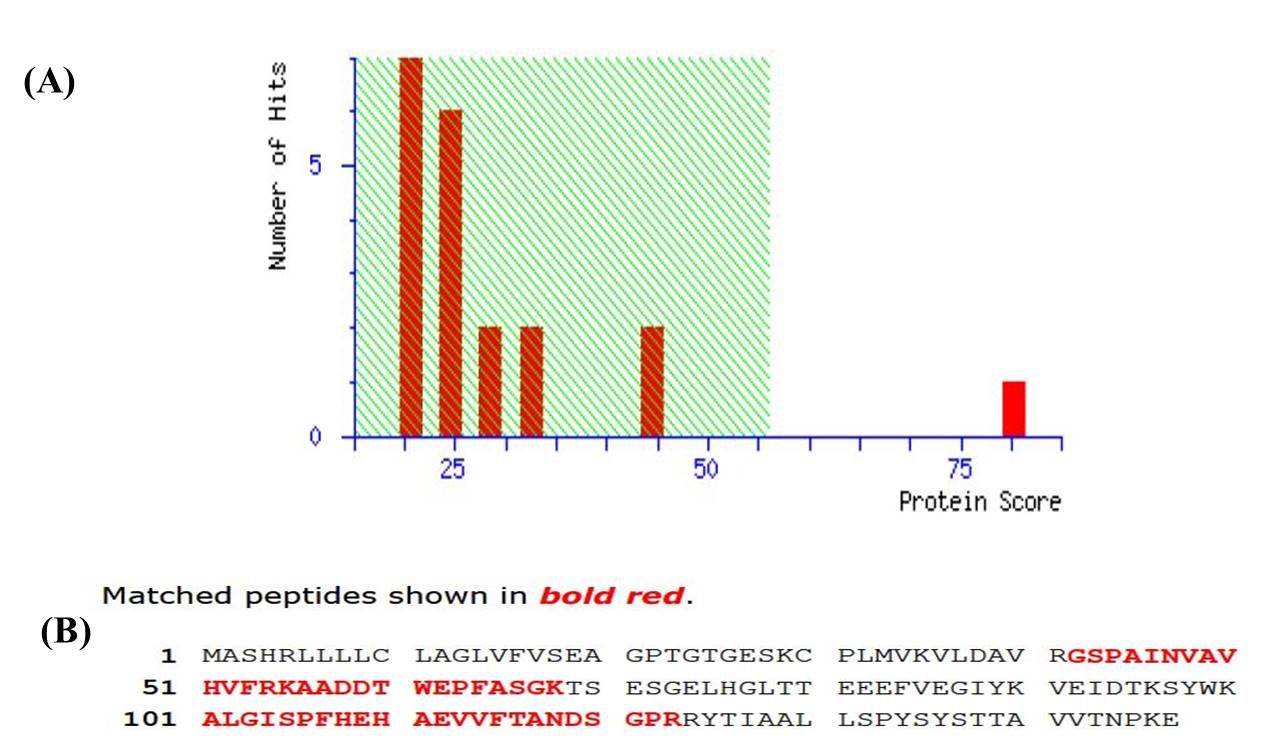


**Supplementary Figure 1. Representative graph of MALDI Mascot score distribution: (A)** The histogram Score distribution showing TTR protein Score with significance (p≤0.05) with protein scores plotted against number of hits (max. 20). With E-value of 0.00019, **(B)** The sequence coverage about 34%, the peptides having a highly significant protein score of 80, were found to correspond to human transthyretin (TTR) protein.

**Supplementary Figure**
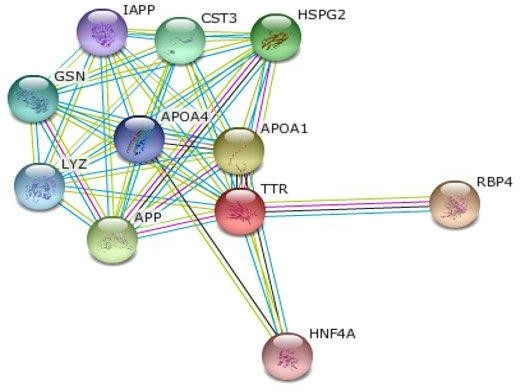
**2**

**Supplementary Figure 2**. **Predicted protein-protein interacting partner of TTR:** The STRING analysis showing the Apolipoprotein A-I (ApoA-I); ApoA-IV; Retinol binding protein (RBP4); Heparan sulfate proteoglycan 2 (HSPG2) and apolipoprotein B (Apo B) that were found to be functional associative interacting proteins partner of TTR.

**Supplementary Figure 3.**


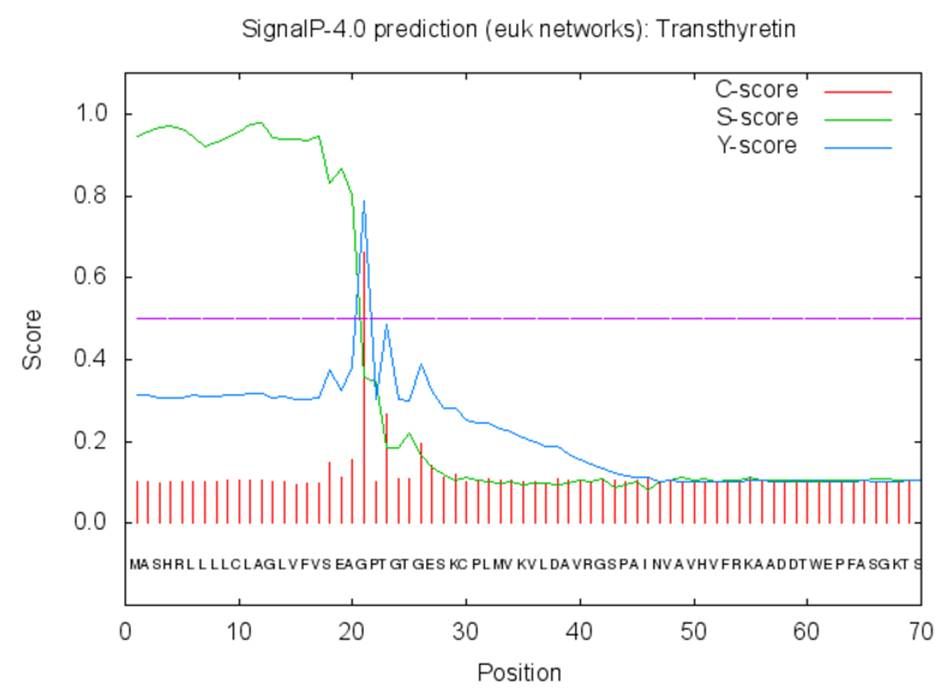


**Supplementary Figure 3. Signal peptide analysis:** Peak indicating the presence of N terminal signal peptide. Peak at 21st position represents the cleavage site of signal peptidases.

**Supplementary Figure 4**


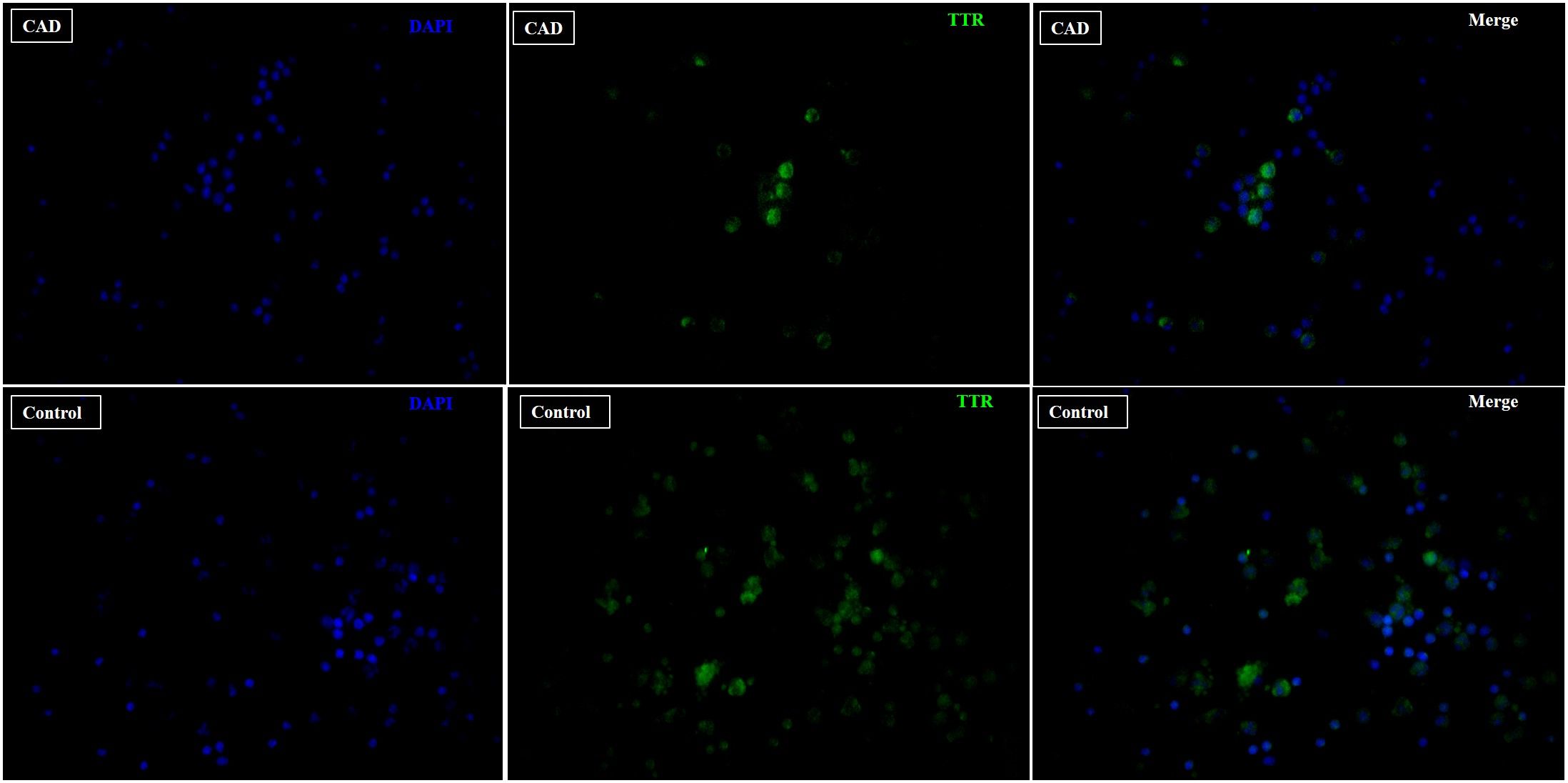


**Supplementary Figure 4. Immunofluorescence analysis of TTR expression and localization**: TTR protein localize within cytosolic region (green shade). The stained TTR protein with Alexa-Flour 546 visualized with depleted levels in CAD patient’s PBMCs compared to healthy control, scale=10μm. 4, 6-diamidino-2-phenylindole (DAPI) was used to stain (blue) the cell nuclei. Original magnification 40x.

**Supplementary Tables**

| **Rank** | **Localization** | **Score** |
| --- | --- | --- |
| 1 | Peroxisome | 0.83 |
| 2 | Cytoplasmic | 0.10 |
| 3 | Extracellular | 0.03 |

**Supplementary Table 1:** Results obtained from MultiLoc server

indicating the probable localization sites of TTR

| **S. No** | **Localization** | **Accuracy** | **Server** |
| --- | --- | --- | --- |
| 1. | Cytoplasm | 60.9% | PSORT II |
| 2. | Cytoskeleton | 13% | PSORT II |
| 3. | Nucleus | 8.7% | PSORT II |
| 4. | Cytoplasm | 56% | SubLoc |

**Supplementary Table 2:** Results indicated the site of localization of TTR predicted through PSORT II and SubLoc servers.
